# The efficacy and safety of first-line therapies for preventing chronic post-surgical pain: a network meta-analysis

**DOI:** 10.18632/oncotarget.22611

**Published:** 2017-11-03

**Authors:** Jie Ning, Jing Luo, Zengdong Meng, Chong Luo, Gang Wan, Jie Liu, Sanrong Wang, Xingye Lian, ND Melgiri, Yang Sun, Rongzhong Huang

**Affiliations:** ^1^ Department of Pain Medicine, The First People's Hospital of Yunnan Province, Yunnan, Kunming, China; ^2^ Department of Orthopedics, The First People's Hospital of Yunnan Province, Yunnan, Kunming, China; ^3^ Department of Gerontology, The Second Affiliated Hospital of Chongqing Medical University, Chongqing, Jiangbei, China; ^4^ Department of Pain Medicine, The Second Affiliated Hospital of Chongqing Medical University, Chongqing, Jiangbei, China; ^5^ Institute of Ultrasound Imaging, The Second Affiliated Hospital of Chongqing Medical University, Chongqing, Jiangbei, China; ^6^ Impactys Foundation for Biomedical Research, San Diego, CA, United States of America

**Keywords:** chronic post-surgical pain, CPSP, nefopam, mexiletine, pregabalin

## Abstract

**Background:**

Due to conflicting evidence regarding first-line therapies for chronic post-surgical pain (CPSP), here we comparatively evaluated the efficacy and safety of first-line therapies for the prevention of CPSP.

**Materials and Methods:**

MEDLINE, EMBASE, and Cochrane CENTRAL databases were searched for randomized, controlled trials (RCTs) of systemic drugs measuring pain three months or more post-surgery. Pairwise meta-analyses (a frequentist technique directly comparing each intervention against placebo) and network meta-analyses (a Bayesian technique simultaneously comparing several interventions via an evidence network) compared the mean differences for primary efficacy (reduction in all pain), secondary efficacy (reduction in moderate or severe pain), and primary safety (drop-out rate from treatment-related adverse effects). Ranking probabilities from the network meta-analysis were transformed using surface under the cumulative ranking analysis (SUCRA). Sensitivity analyses evaluated the impact of age, gender, surgery type, and outlier studies.

**Results:**

Twenty-four RCTs were included. Mexiletine and ketamine ranked highest in primary efficacy, while ketamine and nefopam ranked highest in secondary efficacy. Simultaneous SUCRA-based rankings of the interventions according to both efficacy and safety revealed that nefopam and mexiletine ranked highest in preventing CPSP. Through the sensitivity analyses, gabapentin and ketamine remained the most-highly-ranked in terms of efficacy, while nefopam and ketamine remained the most-highly-ranked in terms of safety.

**Conclusions:**

Nefopam and mexiletine may be considered as first-line therapies for the prevention of CPSP. On account of the paucity of evidence available on nefopam and mexiletine, gabapentin and ketamine may also be considered. Venlafaxine is not recommended for the prevention of CPSP.

## INTRODUCTION

Chronic (or persistent) post-surgical pain (CPSP) is a pain syndrome characterized by pain at the surgical site that continues at least two months post-surgery after all other etiologies have been excluded (e.g., chronic infection, malignancy, etc.) [1]. The most popular first-line therapies for CPSP are gabapentinoids (namely pregabalin and gabapentin) and ketamine [2, 3]. Gabapentinoids have been conventionally used as adjuncts in acute postoperative analgesia, and several previous meta-analyses have demonstrated that they can reduce opioid consumption while improving pain scores [2, 4–6]. A more recent 2012 meta-analysis by Clarke et al. on the use of gabapentinoids for the prevention of CPSP showed that both gabapentin and pregabalin therapy demonstrated significant reductions in CPSP [7]. However, in contrast to Clarke et al.'s findings, Chaparro et al.'s meta-analysis showed a significant reduction in the incidence of CPSP following treatment with ketamine at three months and six months after surgery but showed no such effect with either gabapentin or pregabalin [8].

Thus, there is conflicting evidence regarding the efficacy of gabapentinoids and ketamine for the prevention of CPSP. Moreover, although literature regarding serotonin-norepinephrine reuptake inhibitor (SNRI) use for the prevention of CPSP is limited, the SNRI venlafaxine has also shown promising results [9]. To comprehensively address the use of these agents in preventing CPSP, a Bayesian network meta-analytical approach to mixed treatment comparisons is capable of combining direct evidence and indirect evidence for pairwise comparisons, thereby synthesizing a greater share of the available evidence than conventional meta-analysis [10]. Therefore, the aim of this network meta-analysis was to comparatively evaluate the efficacy and safety of first-line therapies for the prevention of CPSP.

## MATERIALS AND METHODS

### Research question

This study was conducted in accordance with the Preferred Reporting Items for Systematic reviews and Meta-Analyses (PRISMA) Extension Statement for Reporting of Systematic Reviews Incorporating Network Meta-analyses of Health Care Interventions [11, 12]. The research question was structured according to the PICO (Population, Intervention, Comparator, and Outcome) model as follows [13]. The population under study was adult participants of both genders (18 years of age and older) undergoing planned surgical procedures involving tissue injury. The interventions under study were one or more of the following pain medications (i.e., pregabalin, gabapentin, ketamine, or venlafaxine) administered systemically before, during or after surgery, or all. The comparators under study were another pain medication and/or placebo. The efficacy outcomes under study were all pain (as well as moderate or severe pain (at least 4/10)) measured three months or more after surgery using a validated pain assessment instrument such as the Visual Analog Scale (VAS), the Numeric Rating Scale (NRS), the McGill Pain Questionnaire (MPQ), the Neuropathic Pain Diagnostic Questionnaire (DN), and the Leeds Assessment of Neuropathic Symptoms and Signs pain scale (SLANSS) [14]. The safety outcome under study was the drop-out percentage due to treatment-related adverse effects.

### Search strategy

We identified English studies relevant to our network meta-analysis by performing a comprehensive search of the MEDLINE, EMBASE, and Cochrane CENTRAL databases published up to April 2017. The following search strategy was applied: (pregabalin OR gabapentin OR ketamine AND venlafaxine) AND (post-op* OR postop* OR post-surg* OR postsurg* OR “after op*” OR “follow* op*” OR “after surg*” OR “follow* surg*”) AND (pain OR analgesi* OR discomfort) AND (chronic* OR constant* OR continu* OR persist* OR long* OR phantom) AND (trial AND placebo AND random* AND double-blind*). Additional reports were identified from the reference lists of retrieved studies and relevant reviews.

### Eligibility criteria

Only (i) randomized, controlled trials (RCTs) of (ii) one or more drugs (i.e., pregabalin, gabapentin, ketamine, or venlafaxine) administered systemically before, during or after surgery, or both, which (iii) measured pain (using a validated pain assessment instrument) three months or more after surgery on (iv) adult participants of both genders (18 years of age and older) undergoing (v) planned surgical procedures involving tissue injury were included in the meta-analysis. The drugs could be systemically administered immediately before, during, or after the procedure by any dose, route, or frequency.

We excluded (i) non-RCTs (e.g., reviews, case reports/series, clinical observations, long-term safety studies, etc.), (ii) studies administering drugs non-systemically (i.e., subcutaneous delivery), (iii) studies administering gabapentin enacarbil or (S)-ketamine, and (iv) studies in which the results of CPSP patients could not be segregated from patients with other types of pain.

### Data extraction and outcome measures

Two reviewers independently assessed studies for eligibility and extracted the data from each RCT using a standardized data extraction form. Disagreements were resolved through discussion with a third author. The following parameters were extracted from each RCT: first author, year of publication, country of study, study design, number of patients per treatment arm (n), patient age (mean/median ± standard deviation [SD]), patient sex (male/female %), drug regimen (name, dose, route, timing [hours before/after surgery], and duration), type of surgical procedure, number and proportion (%) of patients with follow-up at three months or more post-surgery, and number and proportion (%) of patients for three outcomes: (i) the primary efficacy outcome -- proportion of participants reporting any pain at the anatomical site of the procedure or pain referred to the surgical site, or both (for example phantom limb pain, shoulder pain referred from the diaphragm, etc.) three months or more after the procedure; (ii) the secondary efficacy outcome – proportion of participants reporting moderate or severe pain (at least 4/10) at the anatomical site of the procedure or pain referred to the surgical site, or both (for example phantom limb pain, shoulder pain referred from the diaphragm, etc.) three months or more after the procedure; and (iii) the primary safety outcome – proportion of participants dropping out of the study due to treatment-related adverse effects.

### Cochrane risk of bias assessment

All included RCTs were graded for risk of bias using the Cochrane Risk of Bias tool [15]. The tool classifies key study items into bias categories (e.g., selection bias, performance bias, etc.), which are subject to a risk-of-bias assessment of ‘high’, ‘low’, or ‘unclear’ [16]. As the assessment of a study's internal validity requires adequate reporting, the Cochrane Risk of Bias tool deems risk of bias to be ‘unclear’ when reporting of a particular item is inadequate [17].

### Statistical analysis

WinBUGS version 1.4.3 was used to perform a random-effects Bayesian network meta-analysis [23]. The WinBUGS code used has been provided (Supplementary Information, Appendix 1). Briefly, a random-effects Bayesian network meta-analysis employs an evidence network to conduct multiple pairwise comparisons between interventions, wherein μ_*jb*_ represents the outcome for intervention *b* in study *j*, and δ_*jbk*_ represents the trial-specific differential effect of intervention *k* relative to intervention *b* that follows a normal distribution N(*d_bk_*, σ^2^) as follows [10, 19]:

njk={μjbμjb+δjbkb=A,B,C,k=B,C,D,if k=bif k is'after'bδjbk∼N(dbk, σ2)=N(dAk−dAb, σ2)dAA=0

For the reader's reference, excellent descriptions of the random-effects Bayesian model (with associated mathematical formulae) have been provided by Hoaglin et al. and Higgins et al. [10, 19]. WinBUGS employs a Markov chain Monte Carlo (MCMC) technique, a statistical technique for estimating (by simulation) the expectation of a statistic in a complex model. Briefly, WinBUGS initially assigns the model's parameters some starting arbitrary values and then updates these parameters each iteration using a stochastic process [24]. In this manner, the parameters (samples) generated with each iteration are correlated with the samples from the previous iteration, forming a ‘Markov chain’ [24]. Eventually, this Markov chain provides a ‘converged’ estimate of the model [24]. For the reader's reference, an excellent description of MCMC has been provided by Gilks et al. [25]. Here, the first 50,000 iterations (termed “burn-in”) were discarded, and the results were based on a further set of 100,000 simulations, ensuring that the multiple simulation strings converged. To assure sufficient iterations were generated to achieve convergence, WinBUGS implements the Brooks-Gelman-Rubin convergence diagnostic, which runs several Markov chains with different starting points and compares within-chain and between-chain variance to calculate the Potential Scale Reduction Factor (PSRF) [24]. If the PSRF is close to unity, convergence has be deemed to be achieved [24]. In this study thinning interval was 1, number of chains was 4, and sample size per chain, 100000 for all analyses. Model fit was validated via the residual deviance; if the model fit was adequate, the posterior mean deviance should roughly equate to the number of data points [26]. Non-informative priors were applied for the means’ normal distributions and uniform distributions were applied for SDs. The “placebo’’ treatment strategy was employed as the reference treatment. The relative effects were assessed in terms of a mean difference (MD) with a 95% credibility interval (CrI). The difference between one intervention and placebo or another intervention was deemed to be statistically significant when the MD's 95% CrI did not include zero. The robustness of each network meta-analysis was assessed through comparing the findings against those from the pairwise meta-analyses [27].

Pairwise meta-analyses (comparing each intervention directly against placebo) were performed using a random-effects model based on frequentist methods in Stata 12 (StataCorp LP, College Station, TX, USA) [18]. Briefly, a pairwise meta-analysis directly compares an intervention B with another intervention A, wherein η_*jk*_ represents the outcome for intervention *k* in study *j*, μ_*j*_ represents the outcome for intervention A in study *j*, and δ_*j*_ represents the differential effect of intervention B relative to intervention A in study *j* as follows [10, 19]:

$$\begin{array}{llll} \eta_{\text{jk}} & = & \left\{ \begin{array}{l} \mu_{\text{j}}\\ \mu_{\text{j}}+\delta_{\text{j}} \end{array}\right. & \begin{array}{l} \text{k}=\text{A}\\ \text{k}=\text{B} \end{array}\\ \delta_{\text{j}} & ∼ & \text{N}\left(\text{d},\sigma^{2}\right) & \end{array}$$

A random-effects model based on frequentist methods provides an inference on δ_*j*_'s distribution across all included studies by assuming a normal distribution for δ_*j*_ with a weighted average *d* and heterogeneity variance σ^2^ (as opposed to a fixed-effects model that assumes σ^2^ = 0) [19]. Random-effects models have been deemed superior to fixed-effects models for meta-analyses, as they show lower Type I bias in significance tests for mean effect estimates and interactions and do not overestimate confidence intervals for mean effect estimates [20]. For the reader's reference, excellent descriptions of the random-effects model based on frequentist methods (with associated mathematical formulae) have been provided by Hoaglin et al. and Higgins et al. [10, 19]. The total number of events and the number of patients randomized to each treatment arm were extracted from each included RCT according to the intention-to-treat principle, as clinical efficacy may be overestimated if the intention-to-treat principle is not followed [21]. The relative effects were assessed in terms of a mean difference (MD) with a 95% confidence interval (CI). The difference between each intervention and placebo was deemed to be statistically significant when the MD's 95% CI did not include zero. Inter-study heterogeneity was assessed with the I^2^ statistic [22].

One of the key advantages of the foregoing Bayesian framework is that it can provide rankings of all included interventions [24]. First, probabilities for a particular intervention being ranked at a specific position (first place, second place, etc.) are calculated for each outcome based on their posterior distributions [24]. Then, a cumulative rankogram (alternatively termed a cumulative ranking probability plot) is constructed for each intervention; specifically, a cumulative rankogram for a particular intervention *j* is the plot of the probabilities of intervention *j* assuming each of *T* possible ranks (where *T* is the total number of interventions) [28]. Therefore, the cumulative rankogram presents the overall probability that an intervention would be ranked *n*, where *n* ranges from one to *T* [28]. Then, the surface under the cumulative ranking curve (SUCRA) value – which is a simple transformation of the mean rank *n* that accounts both for the location and variance of the relative treatment effects – is calculated in order to rank the interventions against each other [28]. In essence, the SUCRA value for a particular intervention reports the average proportion of treatments worse than the particular intervention; therefore, the higher the SUCRA value, the superior performance of the intervention for the outcome in question [28]. Publication bias was assessed by funnel plot construction followed by Egger's testing.

To evaluate the impact of age, gender, surgery type, and outlier studies on our conclusions, four sensitivity analyses were performed: age (≥ 50 years vs. < 50 years), gender (≥ 50% male vs. < 50% male), type of surgery (major surgery vs. minor surgery), and omitting the outlier studies on nefopam, mexiletine, and venlafaxine.

## RESULTS

### Included studies

**Figure 1:**
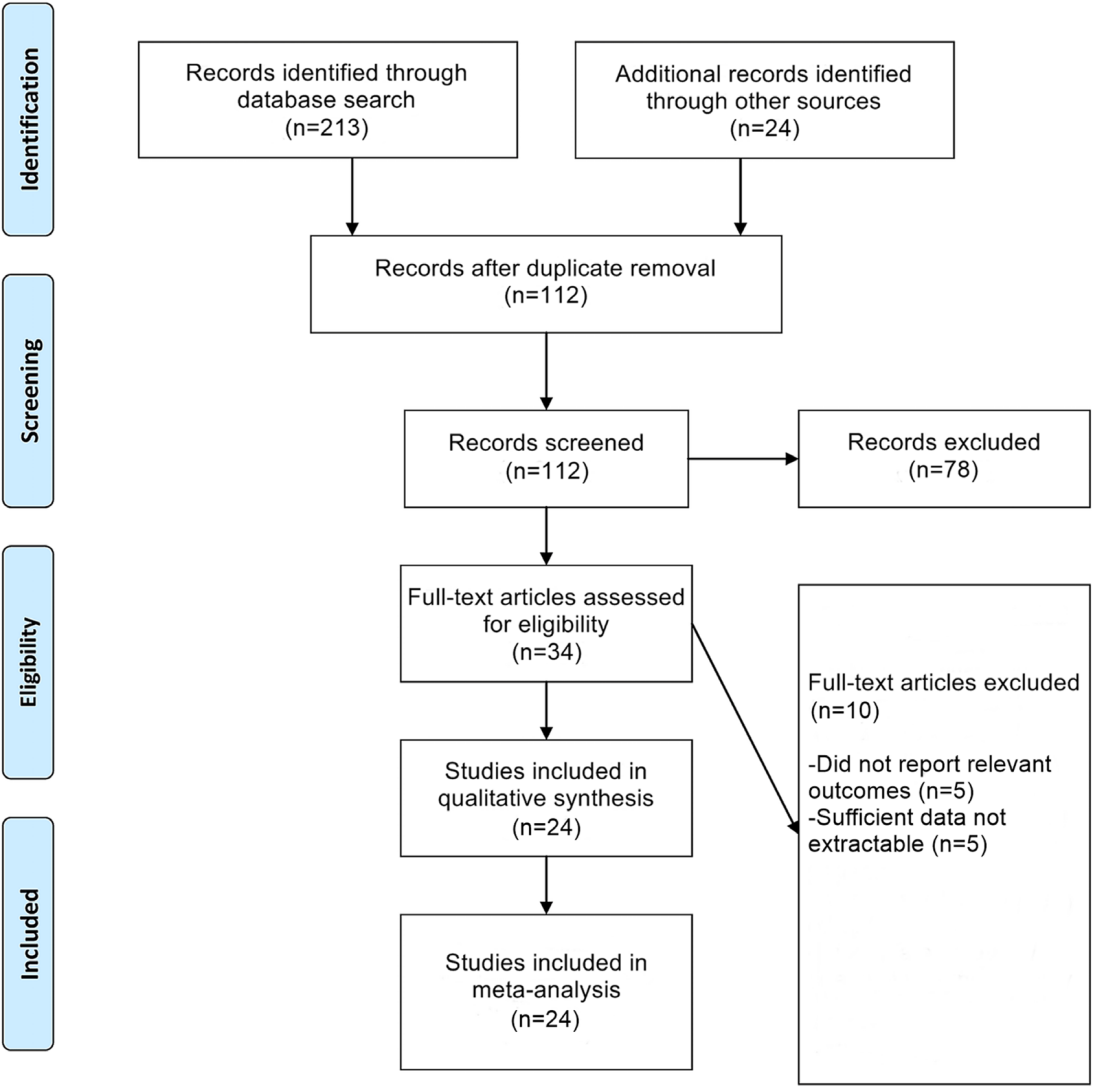
Flowchart of study selection

From an initial set of 237 records, we finally included 24 RCTs in this network meta-analysis (Figure 1) [29–52]. The characteristics of these RCTs have been provided (Supplementary Information, Supplementary Table 1). The Cochrane risk of bias assessments for these RCTs have also been provided (Supplementary Information, Supplementary Table 2). Extracted data from these RCTs enabled us to perform pairwise and network meta-analyses comparing six first-line therapies for preventing CPSP: gabapentin, ketamine, mexiletine, nefopam, pregabalin, and venlafaxine.

### Inter-study heterogeneity, model fit, and inconsistency

The findings from the inter-study heterogeneity analysis revealed a high level of statistical heterogeneity (I^2^ > 75%) for several pairwise comparisons in the primary and secondary efficacy analyses, including gabapentin vs. placebo, ketamine vs. placebo, pregabalin vs. placebo (Supplementary Information, Supplementary Table 3).

Based on the Brooks-Gelman-Rubin statistic, convergence for the network meta-analyses occurred at approximately 6,000 to 8,000 iterations for all outcome measures. The PSRFs were always very close to 1, which means the convergence has been reached. The model's goodness-of-fit to the underlying data, as measured by comparing the number of data points against the posterior mean deviance values, was strong for all outcome measures (Supplementary Information, Supplementary Table 4).

There was no evidence of significant inconsistency in the primary efficacy analysis, as the median MD of 2.07 (95% CrI: 0.10–4.01) did not significantly differ from the median random-effects MD of 0.50 (95% CrI: 0.09–1.28) (*p* > 0.05). Moreover, there was no evidence of significant inconsistency in the secondary efficacy analysis, as the median MD of 1.24 (95% CrI: 0.06–2.41) did not significantly differ from the median random-effects MD of 0.48 (95% CrI: 0.03–1.37) (*p* > 0.05). Finally, there was no evidence of significant inconsistency in the primary safety analysis (median MD of 0.54 (95% CrI: 0.03–1.05) vs. median random-effects MD of 0.54 (95% CrI: 0.03–1.04) (*p* > 0.05).

### Results for primary efficacy outcome

**Figure 2:**
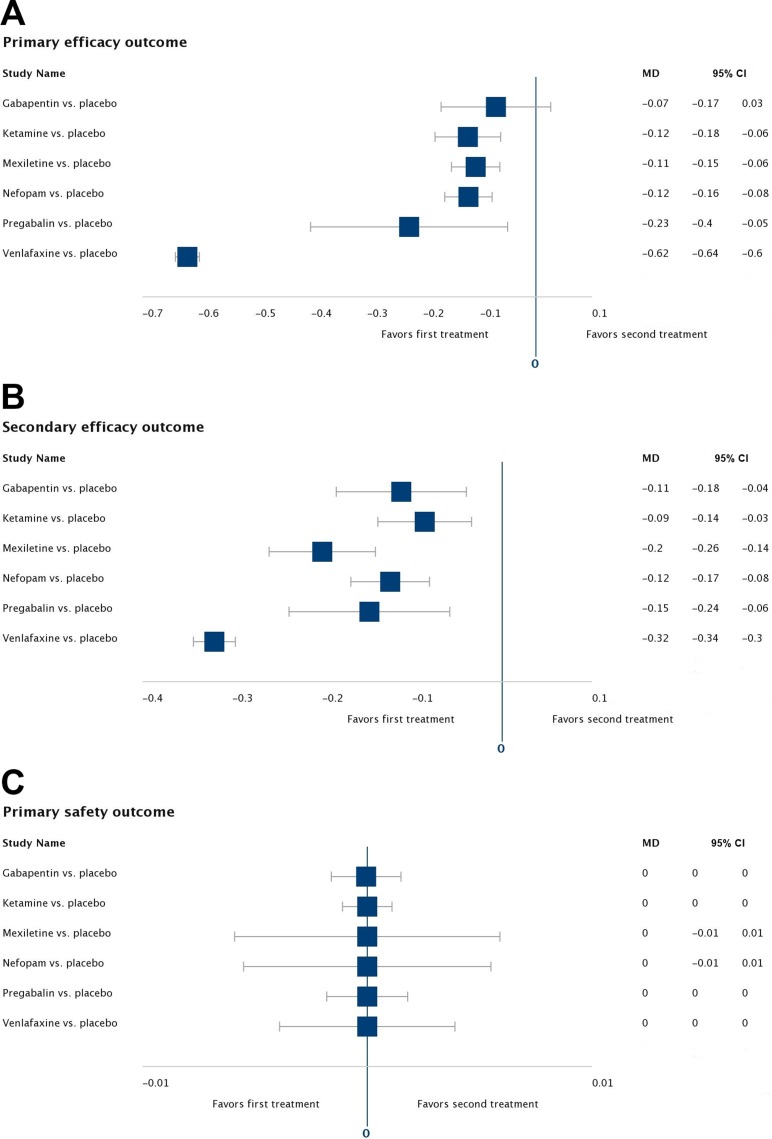
Pairwise meta-analyses for all outcomes Forest plots of the mean differences (MDs) and associated 95% confidence intervals (95% CIs) comparing each treatment against placebo for (**A**) the primary efficacy outcome, (**B**) the secondary efficacy outcome, and (**C**) the primary safety outcome.

**Figure 3:**
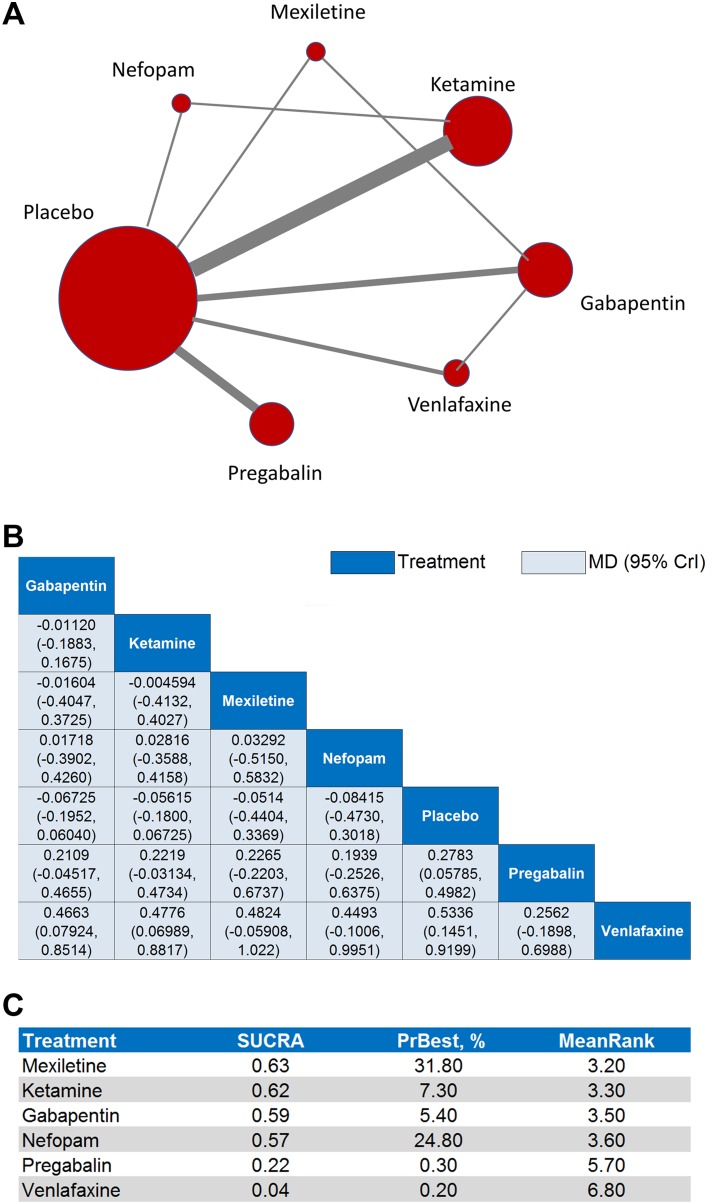
Network meta-analyses for the primary efficacy outcome (**A**) Each node in the network graph represents one intervention included in the primary efficacy network meta-analysis with the size of node reflecting the relative weight. Each solid grey line (edge) between two interventions represents the existence of a direct comparison with the solid grey line's thickness representing the number of studies in each comparison. (**B**) In the league table of point estimates, interventions are displayed along the main diagonal (dark-blue background). The mean difference (MD) and associated 95% credibility interval (CrI) is reported in the cell common between the column-defining intervention and the row-defining intervention (light-blue background). A negative MD value favors the column-defining intervention over the row-defining intervention. (**C**) Surface under the cumulative ranking curve (SUCRA) values, probability of being best (PrBest), and mean ranking (MeanRank) derived from the posterior distributions of all treatments.

The results of the pairwise meta-analysis for the primary efficacy outcome revealed that all six interventions (with the notable exception of gabapentin) were significantly superior to placebo in reducing pain (*p* < 0.05, Figure 2A, Supplementary Information, Supplementary Table 3). The results of the primary efficacy network meta-analysis — network plot (Figure 3A), MD point estimates and associated 95% CrIs (Figure 3B), and SUCRA rankings revealed that mexiletine ranked highest, followed by ketamine and gabapentin, respectively (Figure 3C, Supplementary Information, Supplementary Table 5, Supplementary Figure 1).

The MD point estimates and associated 95% CrIs for the sensitivity analyses on age, gender, surgery type, and omission of the outlier studies have been provided (Supplementary Information, Supplementary Table 6). These sensitivity analyses produced significant changes in the associated SUCRA rankings (Supplementary Information, Table 7); notably, gabapentin ranked first in the age ≥ 50 years, age < 50 years, gender ≥ 50% male, major surgery, and omitting outliers analyses, while ketamine ranked first in the gender < 50% male analysis.

### Results for secondary efficacy outcome

**Figure 4:**
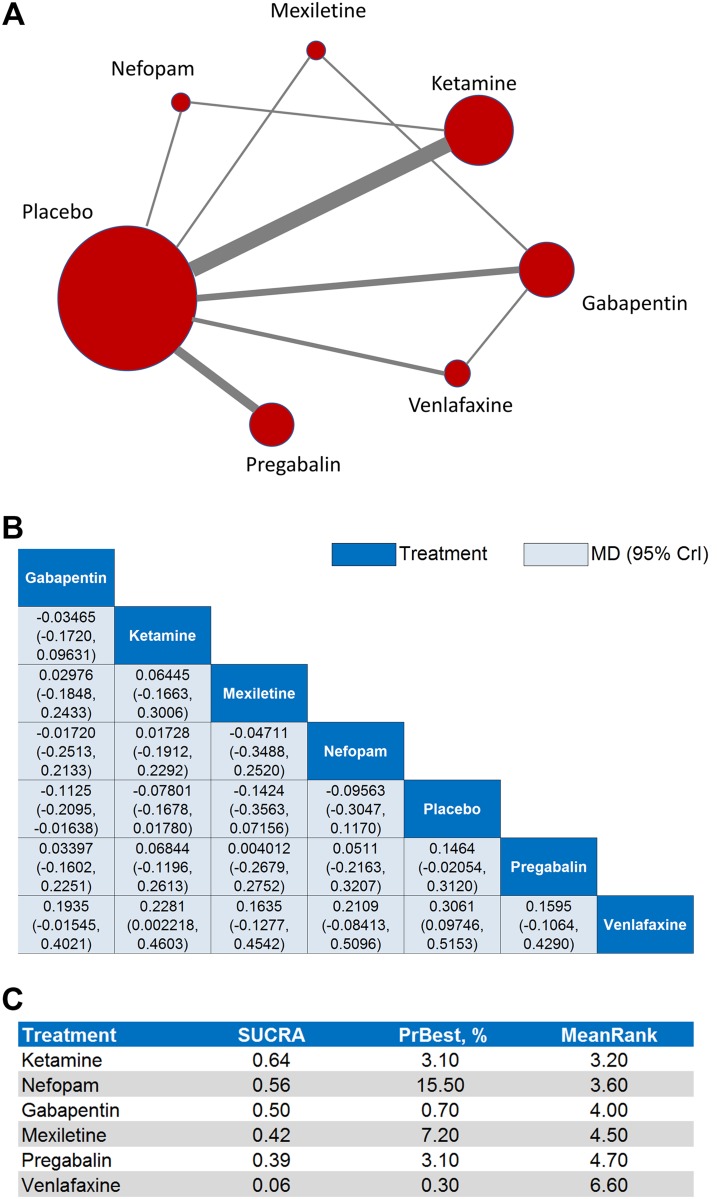
Network meta-analyses for the secondary efficacy outcome (**A**) Each node in the network graph represents one intervention included in the secondary efficacy network meta-analysis with the size of node reflecting the relative weight. Each solid grey line (edge) between two interventions represents the existence of a direct comparison with the solid grey line's thickness representing the number of studies in each comparison. (**B**) In the league table of point estimates, interventions are displayed along the main diagonal (dark-blue background). The mean difference (MD) and associated 95% credibility interval (CrI) is reported in the cell common between the column-defining intervention and the row-defining intervention (light-blue background). A negative MD value favors the column-defining intervention over the row-defining intervention. (**C**) Surface under the cumulative ranking curve (SUCRA) values, probability of being best (PrBest), and mean ranking (MeanRank) derived from the posterior distributions of all treatments.

The results of the pairwise meta-analysis for the secondary efficacy outcome revealed that all six interventions were significantly superior to placebo in reducing moderate or severe pain (*p* < 0.05, Figure 2B, Supplementary Information, Supplementary Table 3). The results of the secondary efficacy network meta-analysis—network plot (Figure 4A), MD point estimates and associated 95% CrIs (Figure 4B), and SUCRA rankings revealed that ketamine ranked highest, followed by nefopam and gabapentin, respectively (Figure 4C, Supplementary Information, Supplementary Table 5, Supplementary Figure 1).

The MD point estimates and associated 95% CrIs for the sensitivity analyses on age, gender, surgery type, and omission of the outlier studies have been provided (Supplementary Information, Supplementary Table 8). These sensitivity analyses produced significant changes in the associated SUCRA rankings (Supplementary Information, Supplementary Table 9); notably, gabapentin ranked first in the age ≥ 50 years, age < 50 years, gender ≥ 50% male, and major surgery, while ketamine ranked first in the minor surgery and omitting outliers analyses.

### Results for primary safety outcome

**Figure 5:**
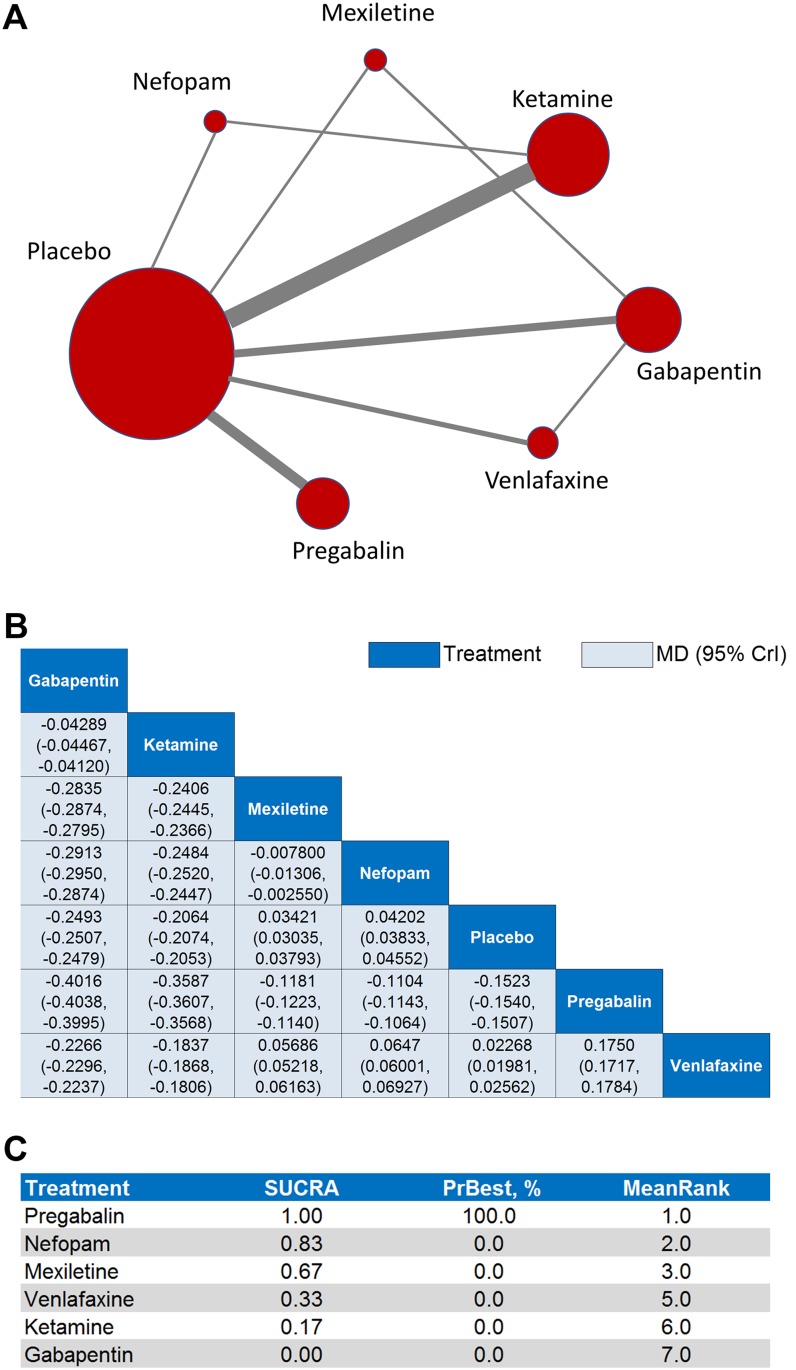
Network meta-analyses for the primary safety outcome (**A**) Each node in the network graph represents one intervention included in the primary safety network meta-analysis with the size of node reflecting the relative weight. Each solid grey line (edge) between two interventions represents the existence of a direct comparison with the solid grey line's thickness representing the number of studies in each comparison. (**B**) In the league table of point estimates, interventions are displayed along the main diagonal (dark-blue background). The mean differences (MD) and associated 95% credibility interval (CrI) is reported in the cell common between the column-defining intervention and the row-defining intervention (light-blue background). A negative MD value favors the column-defining intervention over the row-defining intervention. (**C**) Surface under the cumulative ranking curve (SUCRA) values, probability of being best (PrBest), and mean ranking (MeanRank) derived from the posterior distributions of all treatments.

The results of the pairwise meta-analysis for the primary safety outcome revealed that all six interventions were statistically equivalent to placebo in terms of treatment-related adverse effects (*p* < 0.05, Figure 2C, Supplementary Information, Supplementary Table 3). The results of the primary safety network meta-analysis —network plot (Figure 5A), MD point estimates and associated 95% CrIs (Figure 5B), and SUCRA rankings revealed that pregabalin ranked highest, followed by nefopam and mexiletine, respectively (Figure 5C, Supplementary Information, Supplementary Table 5, Figure 1).

The MD point estimates and associated 95% CrIs for the sensitivity analyses on age, gender, surgery type, and omission of the outlier studies have been provided (Supplementary Information, Supplementary Table 10). These sensitivity analyses produced significant changes in the associated SUCRA rankings (Supplementary Information, Supplementary Table 11); notably, nefopam ranked first in the age < 50 years, gender ≥ 50% male, and minor surgery analyses, while ketamine ranked first in the age ≥ 50 years and omitting outliers analyses.

### Simultaneous SUCRA-based rankings

**Figure 6:**
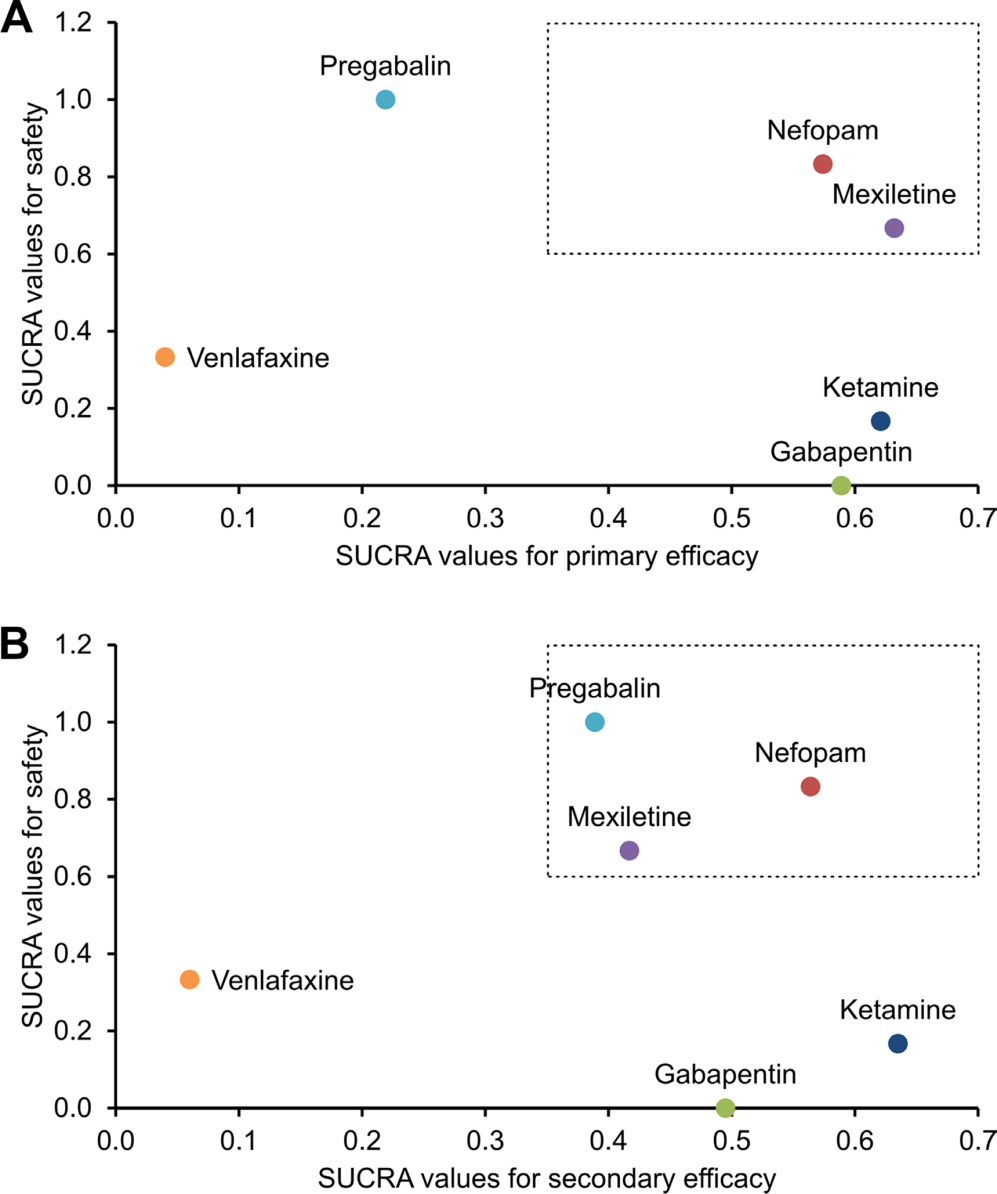
SUCRA-based ranking plots These plots are based on the calculated surface under the cumulative ranking curve (SUCRA) values for (**A**) the primary efficacy and safety outcomes as well as (**B**) the secondary efficacy and safety outcomes. Each colored dot represents a unique treatment. According to the SUCRA valuations, the interventions residing in the upper-right quadrant are more safe and effective than the others.

Simultaneous SUCRA-based rankings of the interventions according to their primary efficacy and safety outcomes revealed that nefopam and mexiletine (occupying the upper-right quadrant) are the safest and most effective interventions for preventing pain in CPSP patients (Figure 6A). Additionally, simultaneous SUCRA-based rankings of the interventions according to their secondary efficacy and safety outcomes revealed that nefopam, mexiletine, and pregabalin (occupying the upper-right quadrant) are the most safe and effective interventions for preventing moderate or severe pain in CPSP patients (Figure 6B). Ketamine and gabapentin (occupying the lower-right quadrants in both plots) were highly effective in preventing CPSP but ranked low on safety (Figure 6A–6B). Venlafaxine (occupying the lower-left quadrants in both plots) was ineffective and ranked low on safety (Figure 6A–6B).

### Publication bias

There was evidence of publication bias in the primary efficacy and secondary efficacy meta-analyses by Egger's testing (*p* = 0.040 and *p* < 0.0001, respectively, Supplementary Information, Supplementary Figure 2). However, there was no evidence of publication bias in the primary safety analysis by Egger's testing (*p* = 0.736, Supplementary Information, Supplementary Figure 2).

## DISCUSSION

Here, we aimed to comparatively evaluate the efficacy and safety of first-line therapies for the prevention of CPSP. Using a Bayesian network meta-analysis of 24 RCTs, we found that nefopam and mexiletine displayed superiority to other first-line therapies in the prevention of all pain in CPSP patients in terms of efficacy and safety. Moreover, nefopam, mexiletine, and pregabalin displayed superiority to other first-line therapies in the prevention of moderate or severe pain in CPSP patients in terms of efficacy and safety. Ketamine and gabapentin were highly effective in preventing CPSP but ranked low on safety, while venlafaxine was relatively ineffective and unsafe. Notably, gabapentin and ketamine remained the most-highly-ranked in terms of efficacy through the various sensitivity analyses, while nefopam and ketamine remained the most-highly-ranked in terms of safety through the various sensitivity analyses.

Nefopam, previously known as fenazoxine, is a benzoxazocine analgesic that is structurally related to the anticholinergic/antihistamine agents orphenadrine and diphenhydramine [53]. Although nefopam is most commonly prescribed for acute postoperative pain [54], it displays pharmacological properties similar to those of NMDA receptor antagonists and monoamine reuptake inhibitors, both of which are used to manage chronic pain [54]. Indeed, a recent network meta-analysis on non-opioid treatments for postoperative pain following major surgery revealed that nefopam plus acetaminophen was superior to all non-opioid analgesic monotherapies in decreasing morphine consumption [55]. Notably, in contrast to other types of analgesics, nefopam has not been shown to affect respiratory function or platelet function [56]. Accordingly, here we found that nefopam was highly effective in preventing CPSP. Moreover, we also found that nefopam had a superior safety ranking relative to mexiletine, ketamine, gabapentin, and venlafaxine, and nefopam also remained the most-highly-ranked intervention in terms of safety following sensitivity analyses. That being said, several serious adverse reactions (such as sweating, tachycardia, nausea, malaise, vomiting, neuropsychiatric side effects, and dermatological side effects) have been reported [53], so clinicians should remain aware of these side effect profiles before prescribing nefopam to CPSP patients.

Mexiletine is a non-selective sodium channel blocker that shows efficacy in reducing pain in painful diabetic neuropathy patients and has been recommended as an orally-available alternative to systemic lidocaine for neuropathic pain syndromes [57, 58]. Moreover, several studies have reported mexiletine's efficacy as an analgesic for non-dystrophic myotonia, a monogenic pain disorder [58]. However, few trials have been performed with specific respect to CPSP prevention. Here, we found that mexiletine was the most highly effective intervention in preventing CPSP. Moreover, we also found that mexiletine had a superior safety ranking relative to ketamine, gabapentin, and venlafaxine. That being said, mexiletine use has been associated with neurological side effects (e.g., nystagmus, blurred vision, dizziness, drowsiness, confused state, tremors, mild ataxia, paresthesia, dysarthria, insomnia, and tinnitus) as well as gastrointestinal symptoms (e.g., nausea, vomiting, anorexia, and dyspepsia) [59, 60]. Clinicians should remain aware of these side effect profiles before prescribing mexiletine to CPSP patients.

Gabapentinoids, such as pregabalin and gabapentin, function by interacting with the accessory α2δ subunits of the voltage-gated gamma-aminobutyric acid (GABA) receptor-channel complex, thereby reducing calcium ion flux through the channel and consequently inhibiting neurotransmitter release [61]. Although their exact mechanism of action in analgesia remains unknown, several hypotheses exist including: altering the expression and trafficking of the α2δ subunit of the voltage-gated GABA receptor-channel complex, suppressing signaling from the central amygdala to the ventrolateral periaqueductal gray area, attenuated stimulus-provoked glutamate release, inhibiting release of substance P, and activating descending noradrenergic inhibitory pain pathways via disinhibiting locus coeruleus neurons [61]. Comparing our current findings to those of conflicting previous meta-analyses regarding pregabalin's use in CPSP, our study concurs with Clarke et al.'s meta-analysis that showed pregabalin's efficacy in reducing CPSP [7]. However, our findings do not support Chaparro et al.'s findings that demonstrated no significant effect for pregabalin upon CPSP [8]. This discrepancy between our work and that of Chaparro et al. may be attributable to inherent differences in the underlying data and analytical approach; Chaparro et al.'s meta-analysis, although well-designed and well-conducted, was restricted to pooling direct pairwise comparisons of pregabalin against placebo from only four trials [8]. The Bayesian network meta-analysis employed here allowed us to comparatively rank pregabalin's efficacy in reducing CPSP against other interventions (and placebo) across a larger network of evidence. Therefore, our current findings lend additional credibility to Clarke et al.'s conclusion that pregabalin is efficacious in reducing CPSP. In addition to the foregoing findings supporting pregabalin's efficacy in preventing CPSP, pregabalin has displayed strong efficacy in post-herpetic neuralgia, diabetic peripheral neuropathy, other forms of neuropathic pain, and fibromyalgia [62]. Moreover, pregabalin also provides significant improvements in patient quality of life (QoL) measures, patient sleep disturbance, and patient- and physician-reported global impression of change measures [62]. With respect to safety, pregabalin ranked higher than all other interventions under study. That being said, pregabalin has been associated with serious adverse effects, such as somnolence, dizziness, dry mouth, peripheral edema, blurred vision, weight gain, changes in mental status, and rhabdomyolysis [61]. Consequently, clinicians should remain aware of these side effect profiles before prescribing pregabalin to CPSP patients.

As opposed to selective serotonin reuptake inhibitors (SSRIs) that selectively function on serotonergic receptors and fail to provide adequate analgesia, SNRIs like venlafaxine display other pharmacological actions that may contribute to their observed analgesic actions, including interference with the opioid system, interaction with NMDA receptors, and inhibition of ion channel activity [63]. Venlafaxine has been shown to be effective in the treatment of painful peripheral diabetic neuropathy, neuropathic pain, fibromyalgia, and migraine prophylaxis [64]. Although venlafaxine is one of the most investigated antidepressant drugs in pain management [64], few trials have been performed with specific respect to CPSP prevention. Here, we found that venlafaxine ranked lower than nefopam, mexiletine, and pregabalin in terms of efficacy in preventing CPSP. Moreover, we also found the venlafaxine had an inferior safety ranking relative to nefopam, mexiletine, and pregabalin; indeed, venlafaxine use has been associated with agitation, diarrhea, increased liver enzymes, hypertension, and hyponatremia [64]. Therefore, based on the current evidence, we do not recommend the use of venlafaxine for the prevention of CPSP.

There are several limitations to this study. First, although nefopam and mexiletine display superiority to other interventions in terms of efficacy and safety, there was only one small RCT directly involving venlafaxine (Amr 2010 [29]), only one small RCT directly involving mexiletine (Fassoulaki 2002 [37]), and only one small RCT directly involving nefopam (Aveline 2014 [30]). Although the Bayesian approach aids in overcoming this paucity of head-to-head trials by enabling indirect comparisons across multiple comparators [10], these small RCTs are still susceptible to bias and insufficient randomization. Therefore, we conducted a sensitivity analysis that specifically omitted the three aforementioned outlier studies; from these analyses, we found that gabapentin and ketamine were strongly favored in terms of efficacy and safety. On this basis, further RCTs on nefopam, mexiletine, and venlafaxine in large, diverse populations of CPSP patients are still required to draw strong conclusions regarding these interventions. Second, in order to adequately power the network meta-analysis, we pooled studies from a wide array of countries consisting of diverse ethnic groups. It is possible that some drugs analyzed here may have differing efficacy and safety profiles in various ethnic groups. Third, this study did not examine the use of combination therapies for the prevention of CPSP; for example, the combination of venlafaxine with gabapentin resulted in a significant additional effect in painful diabetic neuropathy [65]. Fourth, product monographs for the drugs investigated here have reported high rates of adverse effects, while previous reviews have underestimated their adverse effects. Therefore, it is possible that this study may have underestimated the adverse effects of these interventions. Future studies should seek to examine the efficacy and safety profiles of such combination therapies.

In conclusion, based on a Bayesian network meta-analysis of 24 RCTs, nefopam and mexiletine displayed superiority to other first-line therapies in the prevention of all pain in CPSP patients in terms of efficacy and safety. Moreover, nefopam, mexiletine, and pregabalin displayed superiority to other first-line therapies in the prevention of moderate or severe pain in CPSP patients in terms of efficacy and safety. On account of the paucity of evidence available on nefopam and mexiletine, gabapentin and ketamine may also be considered due to their efficacy and safety. The use of venlafaxine is not recommended for the prevention of CPSP. Further trials comparing these agents in large, diverse populations of CPSP patients are needed to validate our findings.

## SUPPLEMENTARY MATERIALS FIGURES AND TABLES




