# Supplementary material for: The efficacy and safety of first-line therapies for preventing chronic post-surgical pain: a network meta-analysis
Source: Oncotarget. 2017 Nov 3;9(62):32081–95. doi: 10.18632/oncotarget.22611 (PMC6112831; doi:10.18632/oncotarget.22611)
Supplement: Supplementary file 1 [file oncotarget-09-32081-s001.pdf]

The efficacy and safety of first-line therapies for preventing chronic post-surgical pain: a network meta-analysis

SUPPLEMENTARY MATERIALS

Supplementary Appendix 1: WinBUGS Code

```
#Random-effects model
model{
        for (i in 1:N.trial){
                prec[i]<- 1/var[i]
                diff[i]~dnorm(delta[i],prec[i])
                delta[i]~dnorm(md[i],tau)
md[i]<- d[t.trial[i]] - d[b.trial[i]]
dev2[i] <- (diff[i]-delta[i])*(diff[i]-delta[i])/var[i]}
        sumdev2 <- sum(dev2[1:N.trial])
 for(i in 1:N.arm){
                prec.y[i]<- n[i]/(sd[i]*sd[i])
                y[i] ~ dnorm(my[i],prec.y[i])
                my[i]<-mu[s[i]]+ delta[i+N.trial]*(1-equals(t.arm[i],b.arm[i]))
                delta[i+N.trial] ~ dnorm(md[i+N.trial],tau)
                md[i+N.trial] <- d[t.arm[i]] - d[b.arm[i]]
        dev[i] <- (y[i]-my[i])* (y[i]-my[i])*prec.y[i]          }
     sumdev <- sum(dev[1:N.arm])
tot.sumdev <- sumdev  + sumdev2
        for(j in 2:53){ mu[j]~dnorm(0,.0001)}
        d[1]<-0
        for (k in 2:NT) {d[k] ~ dnorm(0,.00001) }
        sd.d~dunif(0,50)
                tau<-1/pow(sd.d,2)

        tau.squared <- sd.d*sd.d
# Ranking
        for (k in 1:NT) {
```

Supplementary Table 1: Characteristics of included studies. See Supplementary_Table_1

Supplementary Table 2: Cochrane risk of bias assessments

Study	Random sequence generation (selection bias)	Allocation concealment (selection bias)	Blinding of participants and personnel (performance bias)	Blinding of outcome assessment (performance bias)	Incomplete outcome data (attrition bias)	Selective reporting (reporting bias)	Other bias	Total score
Amr 2010	L	L	L	L	L	L	L	7/7
Aveline 2014	L	L	L	L	L	L	H	6/7
Brogly 2008	L	U	U	U	L	U	H	2/7
Buvanendran 2010	L	L	L	L	L	H	L	6/7
Clarke 2009	L	L	L	U	H	L	H	4/7
Dekock 2001	L	U	L	U	L	L	H	4/7
Duale 2009	U	L	L	L	L	L	H	5/7
Dullenkopf 2009	L	L	L	L	H	L	H	5/7
Fassoulaki 2002	L	L	L	U	L	L	H	5/7
Gianesello 2012	L	L	L	L	L	L	H	6/7
Grosen 2014	L	L	L	L	H	L	L	6/7
Hayes 2004	L	U	L	L	H	L	H	4/7
Katz 2004	L	L	L	L	L	L	L	7/7
Kim 2010	U	U	L	L	L	L	L	5/7
Kinney 2011	U	L	L	L	L	L	L	6/7
Moore 2011	L	L	L	U	H	L	H	4/7
Nikolajsen 2006	L	L	L	L	H	L	H	5/7
Perrin 2009	U	L	L	U	H	L	H	3/7
Pesonen 2011	L	L	L	L	L	L	H	6/7
Remerand 2009	L	L	L	U	L	L	L	6/7
Short 2012	L	U	U	H	U	U	H	1/7
Suzuki 2006	L	U	L	L	L	L	H	5/7
Sveticic 2008	L	U	L	L	H	L	L	5/7
Ucak 2011	L	U	L	U	L	L	H	4/7

We followed the recommended approach for assessing risk of bias in studies included in Cochrane reviews[1]. This tool addresses specific bias domains including methods for generating the random sequence (selection bias), allocation concealment (selection bias), blinding of participants and personnel (performance bias), blinding of outcome assessment (performance bias), incompleteness of outcome data (attrition bias), and selective outcome reporting (reporting bias)[1]. Each item is adjudicated within each study, and the results are represented in the risk of bias table below[1]. The adjudication of the risk of bias is achieved by answering pre-specified questions about the methods reported by each study in relation to the risk domain, such that the conclusion is either low risk of bias (marked "L"), unclear risk of bias (marked "U"), or high risk of bias (marked "H")[1].

Supplementary Table 3: Results of pairwise meta-analyses

Treatment comparison	Pairwise meta–analysis mean difference (MD, [95% CI])	No. of trials	No. of participants	No. of events	Heterogeneity (I^2)
Primary Efficacy Outcome					
Gabapentin vs. placebo	–0.0711 [–0.1687, 0.0265]	10	788	347	99.0%
Ketamine vs. placebo	–0.1210 [–0.1794, –0.0626]	9	1101	159	99.0%
Mexiletine vs. placebo	–0.1071 [–0.1502, –0.0641]	1	45	39	0.0%
Nefopam vs. placebo	–0.1200 [–0.1621, –0.0779]	1	50	9	0.0%
Pregabalin vs. placebo	–0.2255 [–0.4008, –0.0502]	4	469	140	100.0%
Venlafaxine vs. placebo	–0.6200 [–0.6414, –0.5986]	1	100	57	0.0%
Secondary Efficacy Outcome					
Gabapentin vs. placebo	–0.1122 [–0.1845, –0.0398]	6	417	85	97.2%
Ketamine vs. placebo	–0.0861 [–0.1382, –0.0340]	7	410	62	90.8%
Mexiletine vs. placebo	–0.2000 [–0.2591, –0.1409]	1	45	17	0.0%
Nefopam vs. placebo	–0.1245 [–0.1682, –0.0808]	1	50	9	0.0%
Pregabalin vs. placebo	–0.1476 [–0.2370, –0.0582]	2	370	38	95.4%
Venlafaxine vs. placebo	–0.3200 [–0.3433, –0.2967]	1	100	28	0.0%
Primary Safety Outcome					
Gabapentin vs. placebo	0.0000 [–0.0016, 0.0015]	10	662	5	0.0%
Ketamine vs. placebo	0.0000 [–0.0011, 0.0011]	15	1131	0	0.0%
Mexiletine vs. placebo	0.0000 [–0.0059, 0.0059]	1	45	0	0.0%
Nefopam vs. placebo	0.0000 [–0.0055, 0.0055]	1	50	0	0.0%
Pregabalin vs. placebo	0.0000 [–0.0018, 0.0018]	4	469	0	0.0%
Venlafaxine vs. placebo	0.0000 [–0.0039, 0.0039]	1	100	0	0.0%

Supplementary Table 4: Model fit for network meta-analyses

Model	No. of data points	Posterior mean deviance (Dbar)	Leverage (pD)	Deviance information criterion (DIC)
Primary Efficacy	58	61.23917	60.62348	121.86265
Secondary Efficacy	34	33.54163	32.27542	65.81705
Primary Safety	56	63.13142	28.57495	91.70637

If a model's fit is satisfactory, each data point should contribute about one (1.0) to the posterior mean deviance[2]. The sum of the posterior mean deviance and the leverage (also termed the effective number of parameters) is the deviance information criterion (DIC)[2]. The DIC provides an alternative measure of model fit that penalizes model complexity - lower values of the DIC suggest a more parsimonious model[2]. The DIC can be used to compare different models for the same likelihood and data[2].

Supplementary Table 5: Rank probabilities for each treatment

Treatment	Rank						
	[1]	[2]	[3]	[4]	[5]	[6]	[7]
Primary Efficacy Outcome							
Gabapentin	0.0544000	0.1574125	0.2740100	0.3142025	0.1813525	0.0182200	0.0004025
Ketamine	0.0727850	0.1931950	0.2912675	0.2873525	0.1414050	0.0134600	0.0005350
Mexiletine	0.3181150	0.1152050	0.0959375	0.1406850	0.1951450	0.1116300	0.0232825
Nefopam	0.2482750	0.1118500	0.0952175	0.1367325	0.2310325	0.1431625	0.0337300
Placebo	0.3012175	0.4120950	0.2226400	0.0573525	0.0065575	0.0001375	0.0000000
Pregabalin	0.0032600	0.0078700	0.0173800	0.0547050	0.2121550	0.5999300	0.1047000
Venlafaxine	0.0019475	0.0023725	0.0035475	0.0089700	0.0323525	0.1134600	0.8373500
Secondary Efficacy Outcome							
Gabapentin	0.007185	0.103093	0.215410	0.310745	0.259015	0.099545	0.005008
Ketamine	0.030805	0.279455	0.337315	0.213435	0.104495	0.031863	0.002633
Mexiletine	0.072190	0.105493	0.112575	0.139253	0.195230	0.285030	0.090230
Nefopam	0.155485	0.177243	0.162468	0.147513	0.160718	0.152095	0.044480
Placebo	0.701023	0.244405	0.047473	0.006370	0.000680	0.000045	0.000005
Pregabalin	0.030698	0.084935	0.115278	0.164075	0.235870	0.298423	0.070723
Venlafaxine	0.002615	0.005378	0.009483	0.018610	0.043993	0.133000	0.786923
Primary Safety Outcome							
Gabapentin	0.000000	0.000000	0.000000	0.000000	0.000000	0.000000	1.000000
Ketamine	0.000000	0.000000	0.000000	0.000000	0.000000	1.000000	0.000000
Mexiletine	0.000000	0.001848	0.998153	0.000000	0.000000	0.000000	0.000000
Nefopam	0.000000	0.998153	0.001848	0.000000	0.000000	0.000000	0.000000
Placebo	0.000000	0.000000	0.000000	1.000000	0.000000	0.000000	0.000000
Pregabalin	1.000000	0.000000	0.000000	0.000000	0.000000	0.000000	0.000000
Venlafaxine	0.000000	0.000000	0.000000	0.000000	1.000000	0.000000	0.000000

Based on the Bayesian posterior distributions for each intervention, this table details the probabilities for each intervention being ranked first, second, third, etc. for each respective outcome[3].

Supplementary Table 6: Sensitivity analyses for primary efficacy outcome

	Gabapentin	Ketamine	Mexiletine	Nefopam	Pregabalin	Venlafaxine
**Base case**	–0.06725 (–0.1952, 0.06040)	–0.05615 (–0.1800, 0.06725)	–0.0514 (–0.4404, 0.3369)	–0.08415 (–0.4730, 0.3018)	–0.2783 (–0.4982, –0.05785)	–0.5336 (–0.9199, –0.1451)
**Age ≥ 50 years**	0.03119 (–0.1352, 0.1976)	–0.0966 (–0.2167, 0.02223)	-	-	–0.06517 (–0.3023, 0.1705)	-
**Age < 50 years**	–0.09706 (–0.2171, 0.02398)	–0.1377 (–0.2811, 0.002201)	–0.1064 (–0.4076, 0.195)	–0.1284 (–0.4326, 0.1737)	–0.2784 (–0.4749, –0.08179)	–0.5484 (–0.8487, –0.2504)
**Gender ≥ 50% male**	0.0276 (–0.09662, 0.1507)	–0.1334 (–0.2287, –0.04119)	-	–0.1265 (–0.3699, 0.1156)	–0.2775 (–0.4366, –0.1194)	–
**Gender < 50% male**	–0.1675 (–0.3476, 0.01205)	–0.0001703 (–0.4021, 0.3992)	–0.1411 (–0.5038, 0.2205)	-	–0.0657 (–0.4671, 0.337)	–0.5838 (–0.9443, –0.2249)
**Major surgery**	–0.06128 (–0.1693, 0.04642)	–0.1058 (–0.2199, 0.008085)	–0.08848 (–0.3745, 0.1963)	-	–0.2618 (–0.4896, –0.03332)	–0.5307 (–0.8143, –0.2466)
**Minor surgery**	–0.1595 (–0.573, 0.252)	–0.1944 (–0.5126, 0.102)	-	–0.1567 (–0.5516, 0.2248)	–0.1867 (–0.4769, 0.1049)	–
**Omitting outliers***	–0.05378 (–0.1649, 0.05597)	–0.1207 (–0.228, –0.01585)	-	-	–0.2244 (–0.3799, –0.06887)	-

Data reported as mean differences (MDs) with associated 95% credibility interval (CrIs). All MDs use placebo as the comparator intervention.
*The outliers in the evidence network were the individual trials examining nefopam, mexiletine, and venlafaxine.

Supplementary Table 7: SUCRA rankings from sensitivity analyses for primary efficacy outcome

Rank	Base case	Age ≥ 50 years	Age < 50 years	Gender ≥ 50% male	Gender < 50% male	Major surgery	Minor surgery	Omitting outliers*
1	Mexiletine	Gabapentin	Gabapentin	Gabapentin	Ketamine	Gabapentin	Nefopam	Gabapentin
2	Ketamine	Pregabalin	Mexiletine	Nefopam	Pregabalin	Mexiletine	Gabapentin	Ketamine
3	Gabapentin	Ketamine	Nefopam	Ketamine	Mexiletine	Ketamine	Pregabalin	Pregabalin
4	Nefopam	-	Ketamine	Pregabalin	Gabapentin	Pregabalin	Ketamine	-
5	Pregabalin	-	Pregabalin	-	Venlafaxine	Venlafaxine	-	-
6	Venlafaxine	-	Venlafaxine	-	-	-	-	-

Supplementary Table 8: Sensitivity analyses for secondary efficacy outcome

	Gabapentin	Ketamine	Mexiletine	Nefopam	Pregabalin	Venlafaxine
**Base case**	–0.1125 (–0.2095, –0.01638)	–0.07801 (–0.1678, 0.0178)	–0.1424 (–0.3563, 0.07156)	–0.09563 (–0.3047, 0.117)	–0.1464 (–0.312, 0.02054)	–0.3061 (–0.5153, –0.09746)
**Age ≥ 50 years**	–0.0236 (–0.1299, 0.08268)	–0.04735 (–0.1551, 0.06329)	-	-	-	-
**Age < 50 years**	–0.09706 (–0.2171, 0.02398)	–0.1377 (–0.2811, 0.002201)	–0.1064 (–0.4076, 0.195)	–0.1284 (–0.4326, 0.1737)	–0.2784 (–0.4749, –0.08179)	–0.5484 (–0.8487, –0.2504)
**Gender ≥ 50% male**	–0.02344 (–0.1395, 0.093)	–0.08172 (–0.1582, 0.003256)	–	–0.0965 (–0.2752, 0.0858)	–0.147 (–0.2891, –0.003897)	-
**Gender < 50% male**	–0.2026 (–0.375, –0.03574)	–	–0.1874 (–0.4577, 0.08087)	-	-	–0.3513 (–0.6177, –0.08466)
**Major surgery**	–0.08559 (–0.1997, 0.02705)	–0.08449 (–0.198, 0.03017)	–0.1291 (–0.3591, 0.1002)	-	–0.09961 (–0.3524, 0.155)	–0.293 (–0.5193, –0.06702)
**Minor surgery**	–0.2483 (–0.5755, 0.08064)	–0.06521 (–0.2804, 0.2186)	-	–0.09171 (–0.3804, 0.2373)	–0.1913 (–0.518, 0.134)	-
**Omitting outliers***	–0.07903 (–0.2105, 0.05184)	–0.06643 (–0.1736, 0.04929)	-	-	–0.1463 (–0.3301, 0.03836)	-

Data reported as mean differences (MDs) with associated 95% credibility interval (CrIs). All MDs use placebo as the comparator intervention.
*The outliers in the evidence network were the individual trials examining nefopam, mexiletine, and venlafaxine.

Supplementary Table 9: SUCRA Rankings from sensitivity analyses for secondary efficacy outcome

Rank	Base case	Age ≥ 50 years	Age < 50 years	Gender ≥ 50% male	Gender < 50% male	Major surgery	Minor surgery	Omitting outliers*
1	Ketamine	Gabapentin	Gabapentin	Gabapentin	Mexiletine	Gabapentin	Ketamine	Ketamine
2	Nefopam	Ketamine	Mexiletine	Ketamine	Gabapentin	Ketamine	Nefopam	Gabapentin
3	Gabapentin	-	Nefopam	Nefopam	Venlafaxine	Pregabalin	Pregabalin	Pregabalin
4	Mexiletine	-	Ketamine	Pregabalin	-	Mexiletine	Gabapentin	-
5	Pregabalin	-	Pregabalin	-	-	Venlafaxine	Ketamine	-
6	Venlafaxine	-	Venlafaxine	-	-	-	-	-

Supplementary Table 10: Sensitivity analyses for primary safety outcome

	Gabapentin	Ketamine	Mexiletine	Nefopam	Pregabalin	Venlafaxine
**Base case**	–0.2493 (–0.2507, –0.2479)	–0.2064 (–0.2074, –0.2053)	0.03421 (0.03035, 0.03793)	0.04202 (0.03833, 0.04552)	0.1523 (0.1507, 0.154)	–0.02268 (–0.02562, –0.01981)
**Age ≥ 50 years**	–0.03349 (–0.07159, 0.004976)†	3.841e–07 (–0.02351, 0.02349)†	-	–	-	-
**Age < 50 years**	–1.801e–05 (–0.04292, 0.04294)†	–0.01677 (–0.04708, 0.01356)†	-	0.04174 (–0.02435, 0.108)	–1.901e–05 (–0.04276, 0.04289)†	-
**Gender ≥ 50% male**	–5.93e–05 (–0.01902, 0.01899)†	–0.007208 (–0.01964, 0.005273)†	-	0.04679 (0.005941, 0.08752)	9.094e–06 (–0.02676, 0.02679)†	-
**Gender < 50% male**	–0.025 (–0.09009, 0.04015)†	–1.374e–05 (–0.1295, 0.1299)†	–0.01251 (–0.1298, 0.1048)	-	–5.85e–05 (–0.1297, 0.1303)†	0.0376 (–0.08016, 0.1549)
**Major surgery**	1.821e–05 (–0.01885, 0.01894)†	–0.01001 (–0.02566, 0.005732)†	5.828e–06 (–0.04995, 0.04997)	-	–1.357e–07 (–0.03265, 0.03277)†	–0.1012 (–0.1511, –0.0512)†
**Minor surgery**	0.01872 (–0.1244, 0.1628)†	–0.0504 (–0.1509, 0.05145)†	-	0.02476 (–0.109, 0.159)	–0.0001076 (–0.1432, 0.1429)†	-
**Omitting outliers***	0.001227 (–0.02254, 0.02506)†	0.002132 (–0.01583, 0.02019)†	-	-	–1.395e–05 (–0.03358, 0.03346)†	-

Data reported as mean differences (MDs) with associated 95% credibility interval (CrIs). All MDs use placebo as the comparator intervention.
*The outliers in the evidence network were the individual trials examining nefopam, mexiletine, and venlafaxine.
†*P* < 0.05 vs. base case.

Supplementary Table 11: SUCRA rankings from sensitivity analyses for primary safety outcome

Rank	Base case	Age ≥ 50 years	Age < 50 years	Gender ≥ 50% male	Gender < 50% male	Major surgery	Minor surgery	Omitting outliers*
1	Pregabalin	Ketamine	Nefopam	Nefopam	Venlafaxine	Gabapentin	Nefopam	Ketamine
2	Nefopam	Gabapentin	Pregabalin	Gabapentin	Pregabalin	Pregabalin	Gabapentin	Gabapentin
3	Mexiletine	-	Gabapentin	Pregabalin	Ketamine	Mexiletine	Pregabalin	Pregabalin
4	Venlafaxine	-	Ketamine	Ketamine	Mexiletine	Ketamine	Ketamine	-
5	Ketamine	-	-	-	Gabapentin	Venlafaxine	-	-
6	Gabapentin	-	-	-	-	-	-	-

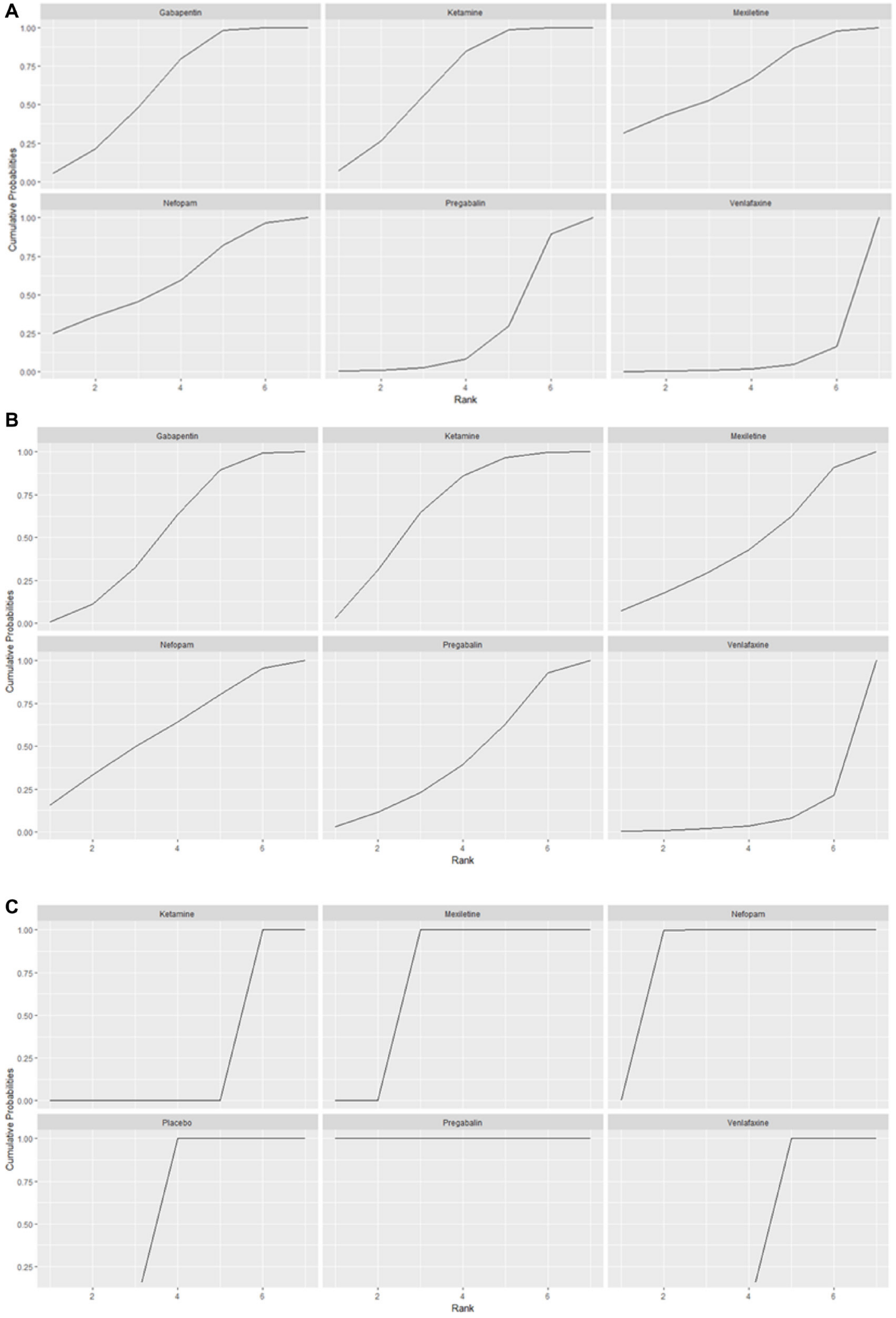

Supplementary Figure 1: Cumulative probability curves for each treatment. cumulative probability curves for (**A**) the primary efficacy outcome, (**B**) the secondary efficacy outcome, and (**C**) the primary safety outcome. The area under the curve (AUC) for the cumulative probability curves can reach a maximum value of 1.00 if the intervention is the best for that event type or a minimum value of 0.00 if the intervention is the worst for that event type[3].

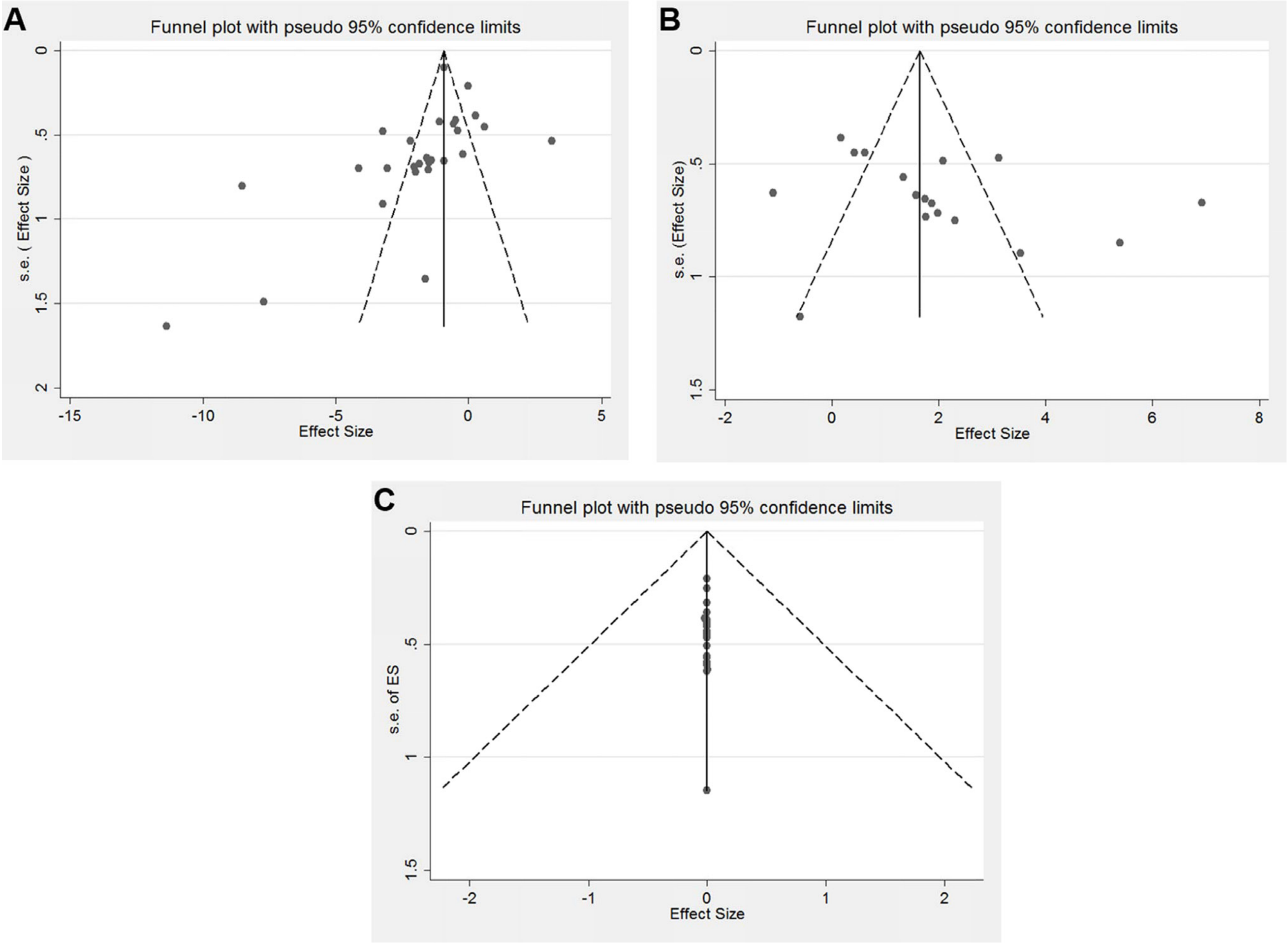

Supplementary Figure 2: Assessment of publication bias. Funnel plots of studies included in (**A**) the primary efficacy outcome analysis, (**B**) the secondary efficacy outcome analysis, and (**C**) the primary safety outcome analysis. Each circle represents one study. The x-axis specifies the effect size (mean difference, MD). The dashed lines represent the triangular region within which 95% of studies are expected to lie in the absence of both bias and heterogeneity. Egger's test was applied to test for the presence of funnel plot asymmetry[4]. For the primary efficacy outcome analysis, the Egger's test ($p = 0.040$) was significant. For the secondary efficacy outcome analysis, the Egger's test ($p < 0.0001$) was significant. For the primary safety outcome analysis, the Egger's test ($p = 0.736$) was not significant.

REFERENCES

1. Higgins JP, Altman DG, Gøtzsche PC, Jüni P, Moher D, Oxman AD, Savovic J, Schulz KF, Weeks L, Sterne JA; Cochrane Bias Methods Group; Cochrane Statistical Methods Group. The Cochrane Collaboration's tool for assessing risk of bias in randomised trials. BMJ. 2011; 343:d5928.
2. Dias S, Sutton AJ, Ades A, Welton NJ. Evidence synthesis for decision making 2: a generalized linear modeling framework for pairwise and network meta-analysis of randomized controlled trials. Medical Decision Making. 2013; 33:607–617.
3. Rücker G, Schwarzer G. Ranking treatments in frequentist network meta-analysis works without resampling methods. BMC medical research methodology. 2015; 15:58.
4. Sterne JA, Sutton AJ, Ioannidis JP, Terrin N, Jones DR, Lau J, Carpenter J, Rücker G, Harbord RM, Schmid CH, Tetzlaff J, Deeks JJ, Peters J, et al. Recommendations for examining and interpreting funnel plot asym.metry in meta-analyses of randomised controlled trials. BMJ. 2011; 343:d4002.
