# Supplementary material for: The efficacy and safety of first-line therapies for preventing chronic post-surgical pain: a network meta-analysis
Source: Oncotarget. 2017 Nov 3;9(62):32081–95. doi: 10.18632/oncotarget.22611 (PMC6112831; doi:10.18632/oncotarget.22611)
Supplement: Supplementary file 2 [file oncotarget-09-32081-s002.docx]

**Supplementary Table 1: Characteristics of Included Studies**

| **Study** | **Country** | **Drug therapy used** | **Patients per treatment arm (n)** | **Patient age (mean/median± SD)** | **Patient sex (M/F %)** | **Dose** | **Route** | **Timing (hours before/after surgery)** | **Duration of drug therapy** | **Type of surgical procedure** | **Number, % of patients follow-up at 3 months or more post-surgery** |
| --- | --- | --- | --- | --- | --- | --- | --- | --- | --- | --- | --- |
| Amr 2010 | Egypt | Venlafaxine | 50 | 45±6 | 0/100 | 37.7 mg/d | Oral | Night before operation | 10 days | Partial or radical mastectomy with axillary dissection | 50, 100% |
|  |  | Gabapentin | 50 | 43±5 | 0/100 | 300 mg/d |  |  |  |  | 50, 100% |
|  |  | Placebo | 50 | 44±8 | 0/100 | - |  |  |  |  | 50, 100% |
| Aveline 2014 | France | Ketamine | 25 | 71±8 | 36.2/73.8 | 0.2 mL/kg + 120 µg/kg/h + 60 µg/kg/h | Infusion | 20 min pre-surgery, at end, second day post-surgery | 48 hours | Total knee arthroplasty | 22, 88% |
|  |  | Nefopam | 25 |  |  | 0.2 mL/kg +  120 µg/kg/h + 60 µg/kg/h |  |  |  |  | 24, 96% |
|  |  | Placebo | 25 |  |  | - |  |  |  |  | 23, 92% |
| Brogly 2008 | France | Gabapentin | 25 | 49 (SD unknown) | 13.6/86.4 | 1200 mg | Oral | 2 hours pre-surgery | Once | Total or partial thyroidectomy | 22, 88% |
|  |  | Placebo | 25 | 49 (SD unknown) | 14.3/85.7 |  |  |  |  |  | 21, 84% |
| Buvanendran 2010 | USA | Pregabalin | 120 | 64±8.3 | 24, 76 | 300 mg (pre), 150 mg*2 (1-10 post), 75 mg*2 (11, 12 post), 50 mg *2 (13, 14 post) | Oral | 1-2 h pre-surgery | 14 days | Total knee arthroplasty | 113, 94.2% |
|  |  | Placebo | 120 | 63.3±8.9 | 30, 70 | - |  |  |  |  | 115, 95.8% |
| Clarke 2009 | Canada | Gabapentin + placebo | 40 | 58.9±9.4 | 67.5, 32.5 | 0 (pre) + 600 mg (post) | Oral | 2 hours pre-surgery | One dose | Total hip arthroplasty | 28, 70% |
|  |  | Placebo + gabapentin | 38 | 60.4±8.1 | 57.9, 42.1 | 600 mg (pre) + 0 (post) |  |  |  |  | 26, 68.4% |
|  |  | Placebo + placebo | 39 | 61.3±10.7 | 53.8, 46.2 | - |  |  |  |  | 28, 71.8% |
| De Kock 2001 | Belgium | Ketamine I | 20 | 65±10 | 45, 55 | 0.25 mg/kg + 0.125 mg/kg/h | Infusion | During surgery | One dose | Resection of rectal adenocarcinoma | 17, 85% |
|  |  | Ketamine II | 20 | 69±6 | 60, 40 | 0.5 mg/kg + 0.25 mg/kg/h |  |  |  |  | 18, 90% |
|  |  | Ketamine III | 20 | 69±6 | 60, 40 | 0.25 mg/kg + 0.125 mg/kg/h |  |  |  |  | 19, 95% |
|  |  | Ketamine IV | 20 | 67±7 | 50, 50 | 0.5 mg/kg + 0.25 mg/kg/h |  |  |  |  | 19, 95% |
|  |  | Placebo | 20 | 67±9 | 50, 50 | - |  |  |  |  | 19, 95% |
| Duale 2009 | France | Ketamine | 42 | 61.9±8.3 | 74, 26 | 1 mg/kg induction, 1 mg/kg/h surgery, 1 mg/kg during 24 h post-surgery | Infusion | During surgery and 24 h post-surgery | 1 day | Thoracotomy | 34, 81% |
|  |  | Placebo | 44 | 58.5±8.5 | 66, 34 | - |  |  |  |  | 35, 79.5% |
| Dullenkopf 2009 | Switzerland | Ketamine I | 36 | 48.6±17.8 | 27.8, 72.2 | 0.15 mg/kg | Infusion | 1 day pre-surgery | 1 day | General and orthopedic surgery | 26, 72.2% |
|  |  | Ketamine II | 41 | 56.2±17.6 | 48.8, 51.2 | 0.5 mg/kg |  |  |  |  | 29, 70.7% |
|  |  | Placebo | 33 | 52.3±17.9 | 45.5, 54.5 | - |  |  |  |  | 25, 75.8% |
| Fassoulaki 2002 | USA | Mexiletine | 21 | 46±5 | 0, 100 | 200 mg*3/d | Oral | Night before operation | 10 days | Partial or radical mastectomy with axillary dissection | 20, 95.2% |
|  |  | Gabapentin | 22 | 42±7 | 0, 100 | 400 mg*3/d |  |  |  |  | 22, 100% |
|  |  | Placebo | 24 | 45±9 | 0, 100 | - |  |  |  |  | 24, 100% |
| Gianesello 2012 | Italy | Pregabalin | 30 | 66.2±10.8 | 30, 70 | 300 mg/d | Oral | 1 h pre-surgery | 2 days | Elective decompressive spine surgery | 30, 100% |
|  |  | Placebo | 30 | 63.5±9.9 | 46.7, 53.3 |  |  |  |  |  | 30, 100% |
| Grosen 2014 | Denmark | Gabapentin | 52 | 67 (SD unknown) | 56, 44 | 1200 mg (pre), 600 mg (1, post), 900 mg (2, post), 1200 mg (3-5, post) | Oral | 2 h pre-surgery | 5 days | Pulmonary malignancy, anterior thoracotomy | 39, 75% |
|  |  | Placebo | 52 | 62 (SD unknown) | 44, 56 | - |  |  |  |  | 37, 71.2% |
| Hayes 2004 | USA | Ketamine | 22 | 68.7±12.2 | 45.5, 54.5 | 0.5 mg/kg (pre), 0.15 mg/kg/h (post) | Infusion | Unknown | 3 days | Above-knee or below-knee amputation | 15, 68.2% |
|  |  | Placebo | 23 | 68.9±10.9 | 69.6, 30.4 | - |  |  |  |  | 17, 73.9% |
| Katz 2004 | Canada | Ketamine + placebo | 47 | 62±5.8 | 100, 0 | 1 mg/ml | Infusion | Before incision, 70 min post-surgery | 1 day | Prostatectomy | 50, 100% |
|  |  | Placebo + ketamine | 50 | 62±6.2 | 100, 0 | 0.0025 ml/kg per min |  |  |  |  | 50, 100% |
|  |  | Placebo + placebo | 46 | 61±6.7 | 100, 0 | - |  |  |  |  | 50, 100% |
| Kim 2010 | Korea | Pregabalin | 50 | 39 (SD unknown) | 93.6, 6.4 | 150 mg | Oral | 1 h pre-surgery | 1 day | Thyroidectomy | 47, 94% |
|  |  | Placebo | 49 | 38 (SD unknown) | 95.7, 4.3 | - |  |  |  |  | 47, 95.9% |
| Kinney 2011 | USA | Gabapentin | 57 | 64.4±7.4 | 56, 44 | 600 mg | Oral | 2 h pre-surgery | 1 day | Thoracotomy | 63, 100% |
|  |  | Placebo | 63 | 64.3±6.8 | 48, 52 | 12.5 mg |  |  |  |  | 57, 100% |
| Moore 2011 | Canada | Gabapentin | 21 | 35±5 | 0, 100 | 600 mg | Oral | 1 h before surgery | 1 day | Cesarean delivery | 16, 76.2% |
|  |  | Placebo | 23 | 34±6 | 0, 100 | - |  |  |  |  | 20, 87.0% |
| Nikolajsen 2006 | Denmark | Gabapentin | 21 | 70.8±11.9 | 52.4, 47.6 | 300 mg first day, 900 mg days 2-4, increased to 2,400 mg/day days 13-30 | Oral | Same day of surgery | 30 days | Lower limb amputation | 15, 71.4% |
|  |  | Placebo | 20 | 69.8±8.5 | 60, 40 | - |  |  |  |  | 18,90% |
| Perrin 2009 | Australia | Ketamine | 5 | 65.6±10.2 | 40, 60 | 0.5 mg/kg | Infusion | Before incision | Unknown | Knee arthroplasty | 5, 100% |
|  |  | Placebo | 7 | 60.3±11.9 | 42.8, 57.2 | - |  |  |  |  | 7, 100% |
| Pesonen 2011 | Finland | Pregabalin | 35 | 79.5 (SD unknown) | 60, 40 | 150 mg | Oral | 1 h before surgery | 6 days | Cardiac surgery | 27, 77.1% |
|  |  | Placebo | 35 | 79.6 (SD unknown) | 45.7, 54.3 | - |  |  |  |  | 30, 85.7% |
| Remerand 2009 | France | Ketamine | 79 | 64±13 | 47, 53 | 0.5 mg/kg | Infusion | Before incision | 1 day | Hip arthroplasty | 72, 91.1% |
|  |  | Placebo | 75 | 65±14 | 55, 45 | - |  |  |  |  | 70, 93.3% |
| Short 2012 | Canada | Gabapentin I | 42 | 34.8±4.1 | 0, 100 | 600 mg | Oral | 1 hour pre-surgery | Once | Cesarean delivery | 112, 88.9% |
|  |  | Gabapentin II | 42 | 35.1±3.8 | 0, 100 | 300 mg |  |  |  |  |  |
|  |  | Placebo | 42 | 35.3±4.8 | 0, 100 | - |  |  |  |  |  |
| Suzuki 2006 | Japan | Ketamine | 24 | 66±14 | 58.3, 41.7 | 0.05 mg/kg per hour | Infusion | During surgery | 3 days | Thoracic surgery | 22, 91.7% |
|  |  | Placebo | 25 | 66±9 | 60, 40 | - |  |  |  |  | 22, 88% |
| Sveticic 2008 | Switzerland | Ketamine | 176 | 48.0±17.2 | 49, 51 | 1.5 mg | Oral | Unknown | Unknown | Orthopedic surgery | 19, 10.8% |
|  |  | Placebo | 176 | 47.3±17.2 | 53, 47 | - |  |  |  |  | 17, 9.7% |
| Ucak 2011 | Turkey | Gabapentin | 20 | 57.4±11.4 | 55, 45 | 1.2 g/d | Oral | 1 hour pre-surgery | 3 days | CABG surgery | 20, 100% |
|  |  | Placebo | 20 | 62.8±10.9 | 65, 35 | - |  |  |  |  | 20, 100% |
